# The Value of Glioma Extent of Resection in the Modern Neurosurgical Era

**DOI:** 10.3389/fneur.2012.00140

**Published:** 2012-10-18

**Authors:** Douglas A. Hardesty, Nader Sanai

**Affiliations:** ^1^Division of Neurological Surgery, St. Joseph’s Hospital and Medical Center, Barrow Neurological InstitutePhoenix, AZ, USA

**Keywords:** glioma, extent of resection, low-grade glioma, high-grade glioma, malignant transformation

## Abstract

**Objective**: There remains no general consensus in the neurosurgical oncology literature regarding the role of extent of glioma resection in improving patient outcome. Although the value of resection in establishing a diagnosis and alleviating mass effect is clear, there is less certainty in ascertaining the influence of extent of resection (EOR). Here, we review the recent literature to synthesize a comprehensive review of the value of extent of resection for gliomas in the modern neurosurgical era. **Methods**: We reviewed every major peer-reviewed clinical publication since 1990 on the role of EOR in glioma outcome. **Results**: Thirty-two high-grade glioma articles and 11 low-grade glioma articles were examined in terms of quality of evidence, expected EOR, and survival benefit. **Conclusion**: Despite limitations in the quality of data, mounting evidence suggests that more extensive surgical resection is associated with longer life expectancy for both low- and high-grade newly diagnosed gliomas.

## Introduction

Gliomas are the most common primary malignant brain tumor in adults, yet outcomes from this aggressive neoplasm remain dismal. In the modern era, the multimodal management of these tumors begins with microsurgical resection whenever possible. Advances in intraoperative technique, including neuronavigation, intraoperative magnetic resonance imaging, intraoperative ultrasound, stimulation mapping techniques, and fluorescence-guided surgery, have all been developed to maximize tumor resection and minimize surgical morbidity for both low- and high-grade gliomas (Barker et al., 1996; Stummer et al., 2006; Sanai and Berger, 2009, 2010; Kubben et al., 2011).

The elucidation of prognostic factors and treatment options for glioma remains a challenge. Patient age and tumor histology have been identified as reliable predictors of patient prognosis among tumor- and treatment-related variables, and patient functional status can also be predictive of outcome. The use of biomarkers such as IDH1/2 and MGMT-methylation status is increasingly common, although the exact role for such markers in routine clinical management remains undefined. The importance of surgical debulking in obtaining a tissue diagnosis and to alleviate symptoms from mass effect is clear, but a lack of Level I evidence limits certainty in assessing the influence of extent of resection (EOR). Despite significant advances over the last two decades in brain tumor imaging and intraoperative technology, the actual impact of glioma resection in extending tumor-free progression, patient quality of life, and patient overall survival remains unclear. With this in mind, we have examined every major clinical publication from 1990 to 2012 that reports on the role of EOR in glioma outcome. Our objective is to synthesize a comprehensive assessment of EOR in the modern neurosurgical era, as well as to critically examine the quality of evidence in the published literature. Lastly, we aim to identify the magnitude of improvement in patient survival, if any, that can be achieved with the degree of EOR.

## Methods

A literature search of the PubMed database from January 1990 to June 2012 was conducted using the following key words: “high-grade glioma,” “low-grade glioma,” “astrocytoma,” “anaplastic astrocytoma,” “oligodendroglioma,” “oligoastrocytoma,” and “glioblastoma (GBM).”

English-language series including adult patients with hemispheric gliomas were identified. Before analyzing the studies in detail, we conducted additional screening. Small series (<75 patients), or those with incomplete methodological data (such as specification of how EOR was assessed), were eliminated. Also excluded were those studies where EOR was recorded only as a volume of residual tumor, or those studies that only compared resection to biopsy. We excluded series that had both adult and pediatric patients if the adult patients had not been analyzed separately. Studies including pilocytic and gemistocytic astrocytomas, where data regarding patients with these tumors were not separated from other low-grade gliomas, were excluded. Based upon these criteria, we found 32 studies examining EOR for high-grade gliomas and 11 studies examining EOR for low-grade gliomas (Tables 1–6).

**Table 1:** **Volumetric EOR studies in high-grade glioma**.

Study	Tumor grade(s)	Patients	Extent of resection (*N*)	Overall survival		
				Mean survival (months)	Univariate analysis *p* value	Multivariate analysis *p* value
Keles et al. (1999)	IV	107	<25% (25)	8.0	NA	<0.0005
			25–49% (21)	14.2	
			50–74% (18)	15.7	
			75–99% (20)	22.1	
			100% (23)	23.3	
Pope et al. (2005)	IV	110	<20%	27.4	NS	NS
			20–89%	11.1	
			90–99%	17.1	
			100%	22.1	
Lacroix et al. (2001)	IV	416	<98%	8.8	<0.0001	<0.0001
			≥98%	13.0	
Keles et al. (2006)	III	102	0–100%	41.0	NS	NS
Sanai et al. (2011)	IV	500	>77%	12.5	<0.0001	0.004
			>79%	12.8	
			>89%	13.8	
			100%	16	

For these remaining studies, we considered the statistical analysis that affected interpretation of data. This assessment included the identification of potential methodological flaws and information that was omitted from the publication. The methodological parameters we evaluated included choice of statistical tests and adjustment for confounding variables. The specific information that we searched for consisted of discussion of possible sources of bias in selection of cases, number of events (deaths), length of follow-up for patients without events, i.e., survivors, hazard ratios, and confidence intervals. We sought to identify potential confounders underlying predictor variables. For example, it could be possible that all patients in whom a subtotal resection (STR) was achieved underwent adjuvant chemoradiation and that none of those in whom a total resection was achieved underwent such therapy. We also looked for a specific statement of how the multivariate analysis, if any, was conducted. Because none of these studies were prospective randomized trials, we looked for how many patients were in each of the analyzed subsets, to determine if studies were adequately powered to see statistically significant differences.

Preoperative and postoperative tumor volumes, from which the EOR is calculated, have been considered as possible prognostic factors. We therefore identified publications to find studies that included evaluations of preoperative and residual tumor volumes on outcome. High-grade gliomas were in all series defined by the enhancing region on a T1-weighted MRI. For low-grade gliomas, tumor volume and EOR were calculated upon T2-weighted MR imaging. Tumor progression was defined by the radiographic progression of these MRI features. Studies using volumetric tumor analysis were evaluated separately, as this method can dramatically affect EOR calculations (Sanai and Berger, 2008, 2009).

## Results

### High-grade glioma studies to date – an overview

Thirty-two studies were identified between 1990 and 2012 examining EOR and high-grade glioma patient outcomes (Shibamoto et al., 1990; Vecht et al., 1990; Hollerhage et al., 1991; Phillips et al., 1991; Sandberg-Wollheim et al., 1991; Curran et al., 1992; Duncan et al., 1992; Prados et al., 1992; Dinapoli et al., 1993; Huber et al., 1993; Simpson et al., 1993; Jeremic et al., 1994; Warnke et al., 1994; Nitta and Sato, 1995; Barker et al., 1996; Kowalczuk et al., 1997; Keles et al., 1999, 2006; Buckner et al., 2001; Lacroix et al., 2001; Levin et al., 2002; Puduvalli et al., 2003; Tortosa et al., 2003; Lamborn et al., 2004; Brown et al., 2005; Pope et al., 2005; Stark et al., 2005; Ushio et al., 2005; Nomiya et al., 2007). Most, but not all, studies used standard WHO grading criteria; other grading systems were utilized in a minority of reports (Dinapoli et al., 1993; Levin et al., 2002; Stark et al., 2005). Five studies used volumetric analysis to determine EOR; the other 27 studies used a combination of 2D postoperative imaging and surgeon judgment to create categories such as gross total resection (GTR), STR, partial resection (PR), etc.

### High-grade glioma volumetric studies

Among the five volumetric studies, three (*N* = 1,023, range 107–500 patients) demonstrated a significant survival benefit for increasing EOR (Table 1; Keles et al., 1999; Lacroix et al., 2001; Sanai et al., 2011). These three positive studies examined the value of EOR in GBM patients only. GTR (EOR = 100%) was associated with a 2- to 8-month increased survival benefit compared to STRs (EOR = 50–98%), depending on series. Two volumetric studies (*N* = 110 and 102, respectively) showed no patient benefit with increasing EOR, although one study did trend toward increased patient survival with increased EOR despite statistical non-significance (Pope et al., 2005; Keles et al., 2006).

### High-grade glioma non-volumetric studies

Seventeen non-volumetric EOR studies (*N* = 4,992, range 86–949 patients) demonstrated a survival benefit associated with increased EOR using univariate or multivariate analyses (Table 2; Shibamoto et al., 1990; Vecht et al., 1990; Curran et al., 1992; Dinapoli et al., 1993; Simpson et al., 1993; Jeremic et al., 1994; Nitta and Sato, 1995; Barker et al., 1996; Buckner et al., 2001; Lamborn et al., 2004; Brown et al., 2005; Stark et al., 2005; Ushio et al., 2005; Nomiya et al., 2007; Stummer et al., 2008; McGirt et al., 2009a; Oszvald et al., 2012). Due to heterogeneity in EOR classification schemes, however, a more detailed meta-analysis of these series is not possible. Similar to the volumetric studies discussed above, overall survival was (depending on series) 0.9–8.0 months longer following GTR as compared to following STR. Of the 14 studies that utilized multivariate analysis to control for age, performance status, and use of adjuvant therapy, only one such analysis (Curran et al., 1992) subsequently rendered EOR non-significant. All other studies using multivariate analysis found greater EOR to independently be associated with improved survival (Table 2). Of note, four studies found EOR to only be associated with increased patient survival in multivariate analysis, but not univariate analysis. Conversely, 11 non-volumetric studies (*N* = 1800, range 75–357 patients) demonstrated no overall survival benefit associated with increased EOR using either univariate or multivariate analysis (Table 3; Hollerhage et al., 1991; Phillips et al., 1991; Sandberg-Wollheim et al., 1991; Duncan et al., 1992; Prados et al., 1992; Huber et al., 1993; Kowalczuk et al., 1997; Levin et al., 2002; Puduvalli et al., 2003; Tortosa et al., 2003; Oszvald et al., 2012). Potential confounders such as use of chemotherapy and radiation therapy varied widely between publications, but did not differ significantly among patients receiving greater or lesser EOR in any individual series.

**Table 2:** **Positive, non-volumetric studies of EOR in high-grade glioma**.

Study	Tumor grade(s)	Patients	Extent of resection (*N*)	Overall survival		
				Mean survival (months)	Univariate analysis *p* value	Multivariate analysis *p* value
Vecht et al. (1990)	III and IV	243	GTR (15)	NA	NA	0.001
			“Large” (15)	
			“Small” (57)	
			“Decompression” (139)	
			Biopsy (17)	
Shibamoto et al. (1990)	IV	135	GTR + STR (50)	15	<0.05	NA
			PR + Biopsy (85)	11	
Simpson et al. (1993)	IV	645	GTR (123)	11.3	<0.001	<0.0001
			Partial (413)	10.4	
			Biopsy (109)	6.6	
Curran et al. (1992)	III	103	GTR (14)	49.3	0.0023	NS
			PR (58)	49.3	
			Biopsy (31)	18.3	
Dinapoli et al. (1993)	III and IV	346	GTR + STR (246)	NA	0.0375	0.0046
			Biopsy (100)	
Jeremic et al. (1994)	IV	86	GTR + STR (61)	14	0.0000	0.00030
			Biopsy (25)	7.3	
Nitta and Sato (1995)	III and IV	101	GTR (26)	20	<0.01	NA
			STR (36)	12	
			PR (39)	11	
Barker et al. (1996)	IV	222	GTR (28)	NA	<0.001	0.04
			STR (165)	
			Biopsy (13)	
Buckner et al. (2001)	III and IV	275	GTR (99)	NA	0.54	0.0002
			STR (169)	
			Biopsy (92)	
Lamborn et al. (2004)	IV	832	GTR (101)	NA	NA	<0.001
			STR (469)	
			Biopsy (86)	
Brown et al. (2005)	III and IV	124	GTR (49)	NA	NS	<0.001
			STR (53)	
			Biopsy (22)	
Ushio et al. (2005)	IV	105	GTR (35)	20	0.0048	0.018
			PR (57)	14.2	
			Biopsy (13)	8.3	
Stark et al. (2005)	IV	267	GTR (167)	NA	0.014	NA
			STR (80)	
			PR (14)	
Nomiya et al. (2007)	III	170	GTR + STR (76)	62.6	<0.0001	0.0001
			PR + Biopsy (94)	22.9	
Stummer et al. (2008)	IV	243	GTR (122)	16.7	<0.001	0.0009
			STR (121)	11.8	
McGirt et al. (2009a)	III and IV	949	GTR (330)	13	0.001	0.001
			“Near-total” (388)	11	
			STR (231)	8	
Oszvald et al. (2012)*	IV	146	GTR (19)	17.7	<0.0001	<0.05
			STR (26)	16.1	
			PR (35)	11.4	
			Biopsy (66)	4.0	

*Significant for patients aged over 65 years only.

**Table 3:** **Negative, non-volumetric studies of EOR in high-grade glioma**.

Study	Tumor grade(s)	Patients	Extent of resection (*N*)	Overall survival		
				Mean survival (months)	Univariate analysis *p* value	Multivariate analysis *p* value
Huber et al. (1993)	III and IV	163	NA	NA	NS	NS
Hollerhage et al. (1991)	IV	118	NA	NA	NS	NS
Prados et al. (1992)	III	357	NA	NA	NA	NS
Duncan et al. (1992)	III and IV	235	GTR (39)	NA	NS	NS
			STR (121)	
			Biopsy (75)	
Sandberg-Wollheim et al. (1991)	III and IV	171	GTR (59)	13.5	NS	NS
			STR (112)	12.8	
Phillips et al. (1991)	IV	173	GTR (28)	NA	NS	NS
			STR (137)	
			Biopsy (8)	
Kowalczuk et al. (1997)	III and IV	75	GTR (30)	NA	NS	NS
			STR (32)	
			Biopsy (13)	
Levin et al. (2002)	III	92	GTR (20)	NA	NA	NS
			STR (45)	
			Biopsy 25	
Puduvalli et al. (2003)	III	106	GTR (30)	NA	NS	NS
			STR (61)	80.4	
			Biopsy (14)	33.6	
Tortosa et al. (2003)	III	95	GTR (33)	NA	NS	NS
			PR (33)	
			Biopsy (29)	
Oszvald et al. (2012)*	IV	215	GTR (41)	16.5	NS	NS
			STR (43)	15.0	
			PR (70)	13.3	
			Biopsy (61)	7.9	

*Non-significant for patients aged under 65 years only.

### Low-grade glioma studies to date – an overview

Eleven studies between 1990 and 2012 evaluated EOR and patient overall and/or progression-free survival for low-grade gliomas (Philippon et al., 1993; Rajan et al., 1994; Leighton et al., 1997; van Veelen et al., 1998; Nakamura et al., 2000; Shaw et al., 2002; Johannesen et al., 2003; Claus et al., 2005; Yeh et al., 2005; Smith et al., 2008; McGirt et al., 2009a). Histologically, the majority of tumors were low-grade astrocytomas, but oligoastrocytomas and oligodendrogliomas were included as a minority of tumors in 10 studies. Three studies used volumetric analysis to determine EOR; the other eight studies used either two-dimensional postoperative imaging parameters or surgeon judgment.

### Low-grade gliomas – volumetric studies

All three studies to date using volumetric analysis to determine EOR in low-grade glioma patients (*N* = 462, range 90–216 patients) have demonstrated a benefit to increasing EOR in univariate and/or in multivariate analysis (Table 4; van Veelen et al., 1998; Claus et al., 2005; Smith et al., 2008). Five-year overall survival was improved in all studies; median survival and time to malignant progression was not always reported. However, in the most recent report by Smith et al. (2008) greater EOR was also predictive of increased malignant progression-free survival.

**Table 4:** **Volumetric EOR studies in low-grade glioma**.

Study	Patients	Extent of resection (*N*)	5-Year overall survival		
			5-Year survival	Univariate analysis *p* value	Multivariate analysis *p* value
van Veelen et al. (1998)	90	>75% (13)	62%	0.002	0.04
		<75% (59)	18%	
Claus et al. (2005)	156	100% (56)	98.2%	0.05	<0.05
		<100% (100)	92.0%	
Smith et al. (2008)	216	0–40% (21)	NA	NA	<0.001
		41–69% (39)	NA	
		70–89% (55)	NA	
		90–99% (26)	97.0%	
		100% (75)	98.0%	

### Low-grade gliomas – non-volumetric studies

Among the eight non-volumetric studies examining the survival benefit associated with EOR for low-grade glioma patients, seven (*N* = 982, range 82–203 patients) demonstrated a benefit with increasing EOR (Table 5; Philippon et al., 1993; Rajan et al., 1994; Leighton et al., 1997; Nakamura et al., 2000; Shaw et al., 2002; Yeh et al., 2005; McGirt et al., 2009a). Five-year overall survival was markedly increased in all series, increasing from 50–70% in STRs to 80–95% in GTR. Cytoreduction remained a significant predictor in the multivariate analyses of six of the seven positive studies (Table 5). Malignant progression-free survival was improved in patients undergoing GTR in the one series that specifically commented upon malignant progression (McGirt et al., 2009a). One large study (*N* = 992) found no significant difference between gross total and subtotal resections in 5-year patient survival (Table 6; Johannesen et al., 2003). As expected, the use of radiation therapy and chemotherapy varied widely between studies. In seven studies, multivariate analysis adjusted for adjuvant radiation therapy, although, in two reports, 100% of patients received postoperative radiation therapy regardless of EOR. In one study (McGirt et al., 2009a), no patient received postoperative radiation therapy prior to disease progression.

**Table 5:** **Positive low grade non-volumetric studies**.

Study	Patients	Extent of resection (*N*)	5-Year overall survival		
			5-Year survival	Univariate analysis *p* value	Multivariate analysis *p* value
Philippon et al. (1993)	179	GTR (45)	80%	0.0002	<0.01
		STR (95)	50%	
		Biopsy (39)	45%	
Rajan et al. (1994)	82	GTR (11)	90%	<0.05	NS
		STR (30)	52%	
		PR (22)	50%	
		Biopsy (19)	42%	
Leighton et al. (1997)	167	GTR (85)	82%	0.008	0.006
		STR (23)	64%	
Nakamura et al. (2000)	88	Radical (43)	NA	<0.001	<0.001
		Non-radical (45)	
Shaw et al. (2002)	203	GTR (29)	88	0.0116	0.0349
		STR (71)	56	
		Biopsy (103)	71	
Yeh et al. (2005)	93	GTR (13)	92	0.0349	0.016
		STR (71)	52	
		Biopsy (9)	52	
McGirt et al. (2009a)	170	GTR (65)	95%	0.043	0.017
		NTR (39)	80%	
		STR (66)	70%	

**Table 6:** **Negative, non-volumetric studies of EOR in low-grade glioma**.

Study	Patients	Extent of resection (*N*)	5-Year overall survival		
			5-Year survival	Univariate analysis *p* value	Multivariate analysis p value
Johannesen et al. (2003)	993	GTR (173)	NA	NS	NS
		STR (689)	
		Biopsy (131)	

## Discussion

To date, no Level I evidence exists comparing EOR and glioma patient outcome. In the modern neurosurgical era, completion of a randomized, placebo-controlled trial examining high-grade glioma EOR remains unlikely. The literature from the last two decades strongly suggests that increasing EOR improves glioma patient survival. This may be particularly important in the surgical resection of low-grade gliomas, where every volumetric EOR study identified and reviewed here has demonstrated survival benefit. Multiple studies have also found a decrease in malignant progression for these low-grade lesions with greater EOR, suggesting a surgical alteration of tumor biology (Smith et al., 2008; McGirt et al., 2009a). This favorable change in the natural history of these lesions from aggressive resection deserves further investigation.

For high-grade gliomas, there is heterogeneity in the EOR literature. The strongest evidence for EOR improving outcome in GBM comes from the randomized trial of 5-ALA published in 2006 by the ALA-Glioma Study Group (Stummer et al., 2006). Patients undergoing surgery with the use of 5-ALA had improved rates of GTR as well as increased 6-month PFS compared to traditional microsurgical resection; however, because patients were randomized to treatment group (5-ALA vs. none) rather than resection category (GTR vs. subtotal), and because subsequent postoperative care was not universal amongst patients with GTR and STRs, this study is not Level I evidence for EOR alone improving outcome. Nevertheless, an extensive retrospective analysis of the study data by the original authors re-stratifying study patients to GTR or STR groups identified complete resection (GTR) as an independent variable in overall survival upon multivariate analysis (Stummer et al., 2008). The ALA-Glioma Study Group data may therefore be considered Level 2b evidence for increasing EOR improving GBM patient outcome – the highest level of evidence from any EOR publication to date. Despite these findings, several retrospective volumetric studies and numerous non-volumetric studies have shown no benefit to increased EOR in high-grade glioma patients. Nevertheless, the overwhelming majority (11 of 14) of large high-grade glioma series (>200 patients), do show survival benefit with greater EOR (Vecht et al., 1990; Duncan et al., 1992; Prados et al., 1992; Dinapoli et al., 1993; Simpson et al., 1993; Barker et al., 1996; Buckner et al., 2001; Lacroix et al., 2001; Lamborn et al., 2004; Stark et al., 2005; Stummer et al., 2008; McGirt et al., 2009a; Sanai et al., 2011; Oszvald et al., 2012). Thus, it remains possible that most of these negative small series may be underpowered to detect a clinically and statistically significant difference.

Balancing cytoreduction with the risk of neurological morbidity is a common theme in many recent reports. Although no study has directly compared this relationship, retrospective studies demonstrate an association between new postoperative deficit and decreased overall survival or worsened functional outcome (McGirt et al., 2009b; Gulati et al., 2011). Interestingly, several large series have demonstrated no difference in new postoperative neurological deficits between patients undergoing GTR vs. subtotal glioma resection (Stummer et al., 2008; McGirt et al., 2009b; Sanai et al., 2011). However, in an analysis of neurological morbidity provided by the ALA-Glioma Study Group, patients in the ALA group had more frequent deterioration on the NIH-Stroke Scale at 48 h (Stummer et al., 2011). Importantly, techniques such as intraoperative cortical stimulation mapping may help mitigate the risks of aggressive glioma resection in eloquent territories (De Witt Hamer et al., 2012).

Many other critical questions remain unanswered. The effect of EOR for different histological subtypes of tumor must be studied, as tumor biology unquestionably plays a role in determining its response to cytoreduction and, perhaps, its ease of obtaining GTR. Similarly, tumor biomarkers known to predict response to adjuvant therapy or overall survival (such as 1p/19q, IDH1/2, and MGMT-methylation) should also be incorporated into future analyses. Also, it is unclear whether the correlation between EOR and survival holds true for both first-time and recurrent operations in either low- or high-grade patients. Lastly, the efficacy of emerging adjuvant therapies, such as bevacizumab, should also be studied in the context of EOR.

## Conclusion

Our review of all studies in the modern neurosurgical era for both low- and high-grade adult supratentorial gliomas highlights recent literature that associate more extensive microsurgical resection with improved life expectancy. In GBM, this level of evidence reaches Level 2b from retrospective analysis of randomized trial data; most evidence is Level 3 from retrospective case series. In addition to providing greater overall survival, more aggressive resections for low-grade gliomas may also impact the natural history of the disease, favorably altering its risk profile for malignant transformation. A prospective, randomized study using modern intraoperative techniques and standardized perioperative adjuncts remains the gold-standard in settling the controversy, but such a trial may never occur. For the time being, retrospective matched studies, retrospective reviews of randomized trials, or prospective observational trials remain the most practical solutions.

## Conflict of Interest Statement

The authors declare that the research was conducted in the absence of any commercial or financial relationships that could be construed as a potential conflict of interest.
